# Effect of Denosumab on Femoral Periprosthetic BMD and Early Femoral Stem Subsidence in Postmenopausal Women Undergoing Cementless Total Hip Arthroplasty

**DOI:** 10.1002/jbm4.10217

**Published:** 2019-08-14

**Authors:** Hannu T Aro, Sanaz Nazari‐Farsani, Mia Vuopio, Eliisa Löyttyniemi, Kimmo Mattila

**Affiliations:** ^1^ Departments of Orthopaedic Surgery and Traumatology Turku University Hospital and University of Turku Turku Finland; ^2^ Unit of Biostatistics, Department of Clinical Medicine University of Turku Turku Finland; ^3^ Department of Diagnostic Imaging Turku University Hospital Turku Finland

**Keywords:** CLINICAL TRIAL, DENOSUMAB, TOTAL HIP ARTHROPLASTY, POSTMENOPAUSAL OSTEOPOROSIS, PERIPROSTHETIC BONE LOSS, IMPLANT MIGRATION, RADIOSTEREOMETRIC ANALYSIS

## Abstract

Antiresorptive denosumab is known to improve the quality and strength of cortical bone in the proximal femurs of osteoporotic women, but its efficacy in preventing periprosthetic bone loss and reducing femoral stem migration has not been studied in women undergoing cementless total hip arthroplasty. We conducted a single‐center, randomized, double‐blinded, placebo‐controlled trial of 65 postmenopausal women with primary hip osteoarthritis and Dorr type A or B proximal femur anatomy. The patients randomly received subcutaneous injections of denosumab 60 mg or placebo once every 6 months for 12 months, starting 1 month before surgery. The primary endpoint was the change in bone mineral density (BMD) of the proximal femur (Gruen zone 7) at week 48, and the secondary endpoint was stem subsidence measured by radiostereometric analysis (RSA) at week 48. Exploratory endpoints included changes in BMDs of the contralateral hip, lumbar spine and distal radius, serum levels of bone turnover markers, walking speed, walking activity, patient‐reported outcome measures, and radiographic assessment of stem osseointegration. The participants underwent vertebral‐fracture assessment in an extension safety study at 3 years. Denosumab significantly decreased bone loss in the medial femoral neck (zone 7) and increased periprosthetic BMD in the greater trochanteric region (zone 1) and lesser trochanteric region (zone 6). Denosumab did not reduce temporary femoral stem migration. The migration occurred mainly during the settling period (0 to 12 weeks) after implantation of the prosthesis. All of the stems osseointegrated, as evaluated by RSA and radiographs. There were no intergroup differences in functional recovery. Discontinuation of denosumab did not lead to any adverse events. In conclusion, denosumab increased periprosthetic BMD in the clinically relevant regions of the proximal femur, but the treatment response was not associated with any reduction of initial stem migration. © 2019 The Authors. *JBMR Plus* published by Wiley Periodicals, Inc. on behalf of the American Society for Bone and Mineral Research.

## Introduction

Total hip arthroplasty is one of the most commonly performed elective surgical procedures.^1^ The procedure is effective for treatment of disabling hip osteoarthritis. The majority of patients receive cementless implant components in the Unites States and Australia^2^, ^3^ but not in all countries.^4^ The largest group to undergo total hip arthroplasty are women aged 65 to 75 years,^4^ and there is an obvious sex issue due to the altered structure of the proximal femur in postmenopausal women.^5^

Hip osteoarthritis does not protect postmenopausal women from developing concomitant osteoporosis.^6^, ^7^ In cementless total hip arthroplasty, osteoporotic cortical bone^5^, ^8^, ^9^ may pose difficulties in achieving axial and rotational stability of femoral stems. Double‐tapered^10^ and parallel‐sided^11^ femoral stem designs rely on initial press‐fit fixation against cortical bone.^2^ Stability is important for biologic osseointegration and the noncemented stems should preferably not migrate at all after surgery.^12^ However, postmenopausal women with low bone mineral density (BMD),^13^ as elderly patients with hip fractures,^14^ are prone to temporary stem migration. Initial subsidence and rotation does not ultimately prevent stem osseointegration,^14^, ^15^, ^16^ and osseointegrated stems do not develop late mechanical loosening.^15^ Bisphosphonates have failed to reduce femoral stem subsidence in various patient populations,^17^, ^18^ including postmenopausal women.^16^ Postmenopausal women with low BMD also suffer from aggravated early periprosthetic bone resorption,^19^ which may appear radiographically as late bone loss around the osseointegrated femoral stems.^20^ According to the strain‐adaptive remodeling theory,^21^, ^22^ the strongest predictors of bone resorption are low cortical index, low BMD, and large stem size.^23^, ^24^

Antiresorptive denosumab is the first biologic therapy approved for postmenopausal osteoporosis.^25^ Reflecting a fundamentally different mechanism of action,^26^ denosumab has a stronger influence on cortical bone remodeling than alendronate.^27^, ^28^ In the proximal femur of postmenopausal women, osteoporotic cortical bone responds rapidly to denosumab therapy by decreasing intracortical porosity^29^ and increasing bone volume and strength.^30^

We hypothesized that denosumab could have a dual effect in postmenopausal women undergoing cementless total hip arthroplasty, namely by preventing periprosthetic bone resorption (primary endpoint) and thereby reducing the amount of initial femoral stem migration occurring before osseointegration (secondary endpoint). Our target was physically active women with Dorr A‐type or B‐type femur morphology, who will likely benefit from the long‐term endurance of cementless fixation techniques. We excluded females with Dorr C‐type femur morphology. They have osteoporosis^31^ and show an increased risk of periprosthetic fracture if treated with cementless total hip arthroplasty.^32^

## Materials and Methods

### Study design

**Figure 1:**
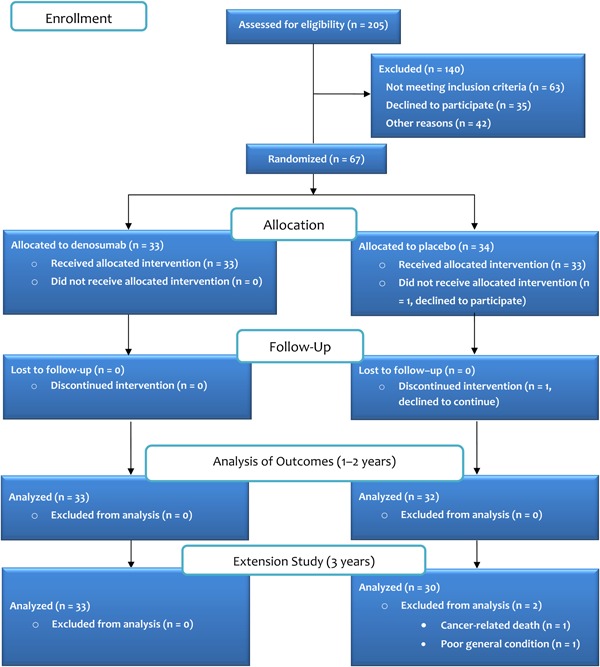
Consolidated Standards of Reporting Trials (CONSORT) flow diagram of the study protocol.

**Figure 2:**
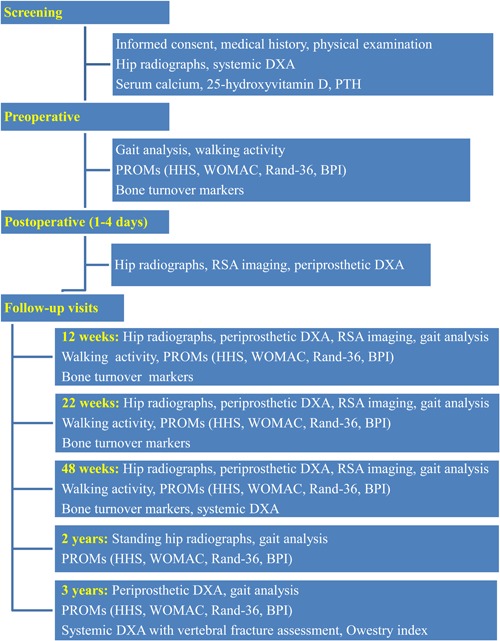
Flowchart of the study assessments and procedures during each visit.

This single‐center randomized, double‐blinded, placebo‐controlled trial evaluated the effects of denosumab in postmenopausal women undergoing cementless total hip arthroplasty. The patients were randomly assigned to receive denosumab or placebo for 1 year (Fig. 1), which covers the phase of periprosthetic bone loss occurring during the first 3 to 12 months after surgery^33^ and the phase of initial femoral stem migration encountered during the settling period of the first 3 to 6 months after surgery.^16^ The clinical outcome of surgery was determined at 2 years, which is the standard time for primary evaluation of functional recovery and early surgical complications after total hip arthroplasty.^34^ The participants were recalled for an extension safety study at 3 years (Figs. 1 and 2).

This study was registered at clinicaltrials.gov (NCT01926158). It was conducted in accordance with the ethical principles of the Declaration of Helsinki. Approval was obtained from the Ethics Committee of the Hospital District of South‐West Finland (decisions 105/2012 and 484/2017) and Finnish Medicines Agency (decision 183/06.00.00/2012, EudraCT 2011‐000628‐14). All study participants provided written informed consent before enrollment.

### Study subjects and screening studies

The subjects were recruited between November 2013 and June 2015 from the patient population of Turku University Hospital admitted for primary total hip arthroplasty. After prescreening using electronic admission data, all potential candidates were assessed for eligibility (Fig. 1). The inclusion criteria were postmenopausal women between 60 and 85 years of age, with incapacitating primary hip osteoarthritis and Dorr A‐type or B‐type femur morphology. The exclusion criteria included severe osteoporosis (hip or lumbar spine *T*‐score < –4.0), Dorr C‐type femur morphology, history of previous surgery of the index hip, evidence of secondary osteoporosis, rheumatoid arthritis or any other inflammatory arthritis, hepatic disease, vitamin D deficiency, disorders of parathyroid function, uncontrolled hyperthyroidism or hypothyroidism, history of malignancy (except basal cell carcinoma of the skin) within the last 5 years, severe asthma or chronic obstructive pulmonary disease, ever use of oral or intravenous bisphosphonates, use of other drugs that affect bone metabolism, Paget's disease, alcohol abuse, or mental, neurological, or other conditions that may affect the ability to perform functional or clinical assessments required by the protocol.

Screening included dual‐energy X‐ray absorptiometry (DXA) of areal BMD (Hologic, Discovery A, Hologic Inc., Marlborough, MA, USA) at the proximal femurs of both hips and lumbar spine (Table 1). Seven patients underwent preoperative DXA of the operated hip only due to the previous total hip arthroplasty of the contralateral hip. As suggested,^35^ the BMD measurement of the distal (one‐third) radius of the nondominant hand was also performed (Table 1). According to the official criteria of the International Society for Clinical Densitometry (ISCD),^36^ the measurement of forearm BMD was not included for the diagnosis of osteoporosis or osteopenia (Table 1). Serum levels of ionized calcium and 25‐hydroxyvitamin D were measured for exclusion of hypocalcemia and vitamin D deficiency, respectively. There were no exclusions due to primary or secondary osteoporosis.

**Table 1:** Baseline Patient Characteristics

	Denosumab	Placebo	*p* Value
Age at consent (years)			
Mean ± SD (*n*)	69.1 ± 5.2 (33)	69.1 ± 5.9 (32)	0.963
Range	61−79	60−84	
BMI (kg/m^2^), mean ± SD (*n*)	27.9 ± 5.3 (33)	28.0 ± 3.6 (32)	0.962
ASA			
Class I–II (no. [%])	17 (51)	22 (69)	0.342
Class III (no. [%])	16 (49)	10 (31)	
History of low‐energy fractures (no. [%])			0.835
Yes	9 (27)	8 (25)	
No	24 (73)	24 (75)	
25‐hydroxyvitamin D (nmol/L), mean ± SD (*n*)	97.6 ± 28.6 (33)	94.0 ± 28.7 (32)	0.614
Operated hip			0.690
Total hip BMD (g/cm^2^), mean ± SD (*n*)	0.91 ± 0.16 (33)	0.92 ± 0.13 (32)	
Femoral neck BMD (g/cm^2^), mean ± SD (*n*)	0.81 ± 0.15 (33)	0.86 ± 0.13 (32)	0.161
Lumbar spine BMD (g/cm^2^), mean ± SD (*n*)	1.01 ± 0.19 (33)	0.98 ± 0.15 (32)	0.452
Distal radius BMD (g/cm^2^), mean ± SD (*n*)	0.64 ± 0.07 (33)	0.66 ± 0.07 (32)	0.272
Low BMD diagnosis (no. [%])^a^			0.343
Normal BMD (*T*‐score ≥ –1.0)	16 (48)	15 (47)	
Osteopenia (–2.5 < *T*‐score < –1.0)	15 (46)	17 (53)	
Osteoporosis (*T*‐score ≤ –2.5)	2 (6)	0 (0)	
Cortical index (mm), mean ± SD (*n*)	9.2 ± 1.6 (33)	9.6 ± 1.6 (32)	0.294
Canal flare index, mean ± SD (*n*)	3.8 ± 0.7 (33)	3.8 ± 0.6 (32)	0.891
Size of the femoral stem, median (range) (*n*)	3 (1–6) (33)	3 (2–5) (32)	0.836
Femoral offset (mm)			
Preoperative, mean ± SD (*n*)	38.2 ± 5.1 (33)	37.8 ± 4.1 (32)	0.701
Postoperative, mean ± SD (*n*)	37.7 ± 5.4 (33)	37.6 ± 4.7 (32)	0.893
Stem‐to‐canal fill ratio			
Proximal stem (%), mean ± SD (*n*)	98.1 ± 2.3 (33)	97.4 ± 2.4 (32)	0.250
Middle stem (%), mean ± SD (*n*)	86.0 ± 7.0 (33)	85.5 ± 9.7 (32)	0.834
Harris hip score, mean ± SD (*n*)	48.1 ± 14.3 (33)	49.0 ± 15.0 (32)	0.794
WOMAC score, mean ± SD (*n*)	46.6 ± 14.3 (33)	48.9 ± 17.4 (30)	0.577
Rand‐36 score			
Physical component, mean ± SD (*n*)	34.5 ± 18.7 (32)	31.6 ± 15.7 (32)	0.503
Mental component, mean ± SD (*n*)	55.5 ± 16.8 (33)	53.1 ± 20.1 (32)	0.610
Walking speed (m/s), mean ± SD (*n*)	0.91 ± 0.25 (32)	0.92 ± 0.28 (31)	0.953
Walking activity^b^ (steps/d), mean ± SD (*n*)	3250 ± 1930 (30)	2910 ± 1910 (30)	0.505
Operation time (minutes), mean ± SD (*n*)	83 ± 11 (33)	81 ± 9 (32)	0.263
Blood loss during surgery (mL), mean ± SD (*n*)	370 ± 150 (33)	350 ± 140 (32)	0.621

BMI = body mass index; ASA = Physical Status Classification of the American Society of Anesthesiologists; BMD = bone mineral density; WOMAC = Western Ontario and McMaster Universities Osteoarthritis Index.

For continuous data, *p* values are from two independent samples *t* test for normally distributed variables or from two independent samples Mann‐Whitney *U* test. For categorical variables, *p* values are from chi‐square test or Fisher's exact test.

^a^ Based on *T*‐scores of the lumbar spine and the hips.

^b^ Pedometer‐measured activity during a 7‐day period before surgery.

### Randomization, intervention, and blinding

Vitamin D and calcium supplementation was started during the screening visit at the minimum of 2 weeks before administration of denosumab/placebo. The subjects were randomized by a computer‐generated random sequence (4Pharma Ltd, Turku, Finland). Stratification based on BMD (*T*‐score < –2.0 or ≥ –2.0) was stopped at an interim analysis owing to the low number of patients with *T*‐score < –2.0.

A clinical dose of 60 mg denosumab every 6 months was selected because it provides the maximal biologic effect at the minimum exposure dose.^25^ The trial subjects received the first subcutaneous dose of denosumab or placebo 1 month before surgery and the second injection at 6 months for the effective treatment period of 1 year.

Patients, investigators, and study personnel, except the coding hospital pharmacy personnel, remained blinded during the study. The investigational products were manufactured and packaged as single‐use prefilled syringes. Placebo was designed and packaged in identical containers as denosumab.

### Surgery and radiographic analysis

Cementless total hip arthroplasty was performed by a single orthopedic surgeon using an anterolateral Hardinge approach. The procedure involved implantation of a parallel‐sided femoral component^11^, ^37^ (Accolade II, Stryker Orthopaedics, Mahwah, NJ, USA), with a metallic head and a porous‐coated acetabular cup with polyethylene liner. The patients were mobilized with use of standard physiotherapy, and unrestricted weight‐bearing was encouraged with the aid of crutches.

A computerized method (Rhinoceros software, version 3.0SR5b, Robert McNeel & Associates, Seattle, WA, USA) was applied to measure femoral offset,^38^ canal flare index,^39^ cortical index,^40^ and stem‐to‐canal fill ratio^41^ from the anteroposterior hip radiographs. Fill ratio was defined as the percentage of endosteal space occupied by the implant.^42^ The ratio of the stem width over the femoral canal width was measured 10 mm above the lesser trochanter (proximal stem) and 60 mm below the lesser trochanter (middle stem).

### Primary endpoint

The primary endpoint was the percentage change from baseline in periprosthetic BMD of Gruen zone 7 of the proximal femur at week 48. DXA measurement for periprosthetic BMD was performed for seven zones within 4 days after surgery (baseline) and at 12, 22, and 48 weeks (Fig. 2). The measured precision^19^ was 1.5% to 3.4%, depending on the zone.

### Secondary endpoint

The secondary endpoint was the three‐dimensional migration of the femoral stem measured by model‐based radiostereometric analysis (RSA)^43^ at week 48. RSA is considered safe.^44^ Baseline RSA imaging was performed within 3 days after surgery and at postoperative 12, 22, and 48 weeks. At each time point, stem migration was determined in relation to the baseline position. Translations and rotations along and around the *x, y, z* axes were measured. Translation along the longitudinal *y* axis (stem subsidence) was selected as the outcome measure. RSA has the highest precision in measurement of this parameter. Stem subsidence has been applied as a predictor of late revision.^45^ Computer‐aided design surface models of each stem size were provided by the implant manufacturer and converted to the model‐based format for calculation of stem 3D migration (MBRSA software version 3.34; Medis Specials BV, Leiden, The Netherlands) by using a combination of stem‐head models. Multiple tantalum RSA markers (1‐mm diameter) were implanted into the trochanteric bone. No stem markers were needed. Stability and adequate distribution of bone markers were assessed by calculating mean error of rigid body fitting (upper limit ≤0.35) and condition number (upper limit ≤150).^46^ The accuracy and precision of model‐based RSA was verified in a pretrial experiment using a phantom model.^43^ Clinical precision for each axis was determined based on double examinations of 58 trial subjects. As recommended,^47^ the clinical precision was calculated as *t* × SD, where *t* is the critical value of the two‐tailed 95% *t* distribution and SD is the standard deviation of the differences between the paired measurements of double examinations. For the measurement of translation, the clinical precision was 140 µm for *x* axis, 110 µm for *y* axis, and 350 µm for *z* axis. For the measurement of rotation, the clinical precision was 0.50 degrees for *x* axis, 1.04 degrees for *y* axis, and 0.18 degrees for *z* axis. Outliers were excluded from this analysis.

### Exploratory and safety endpoints

Exploratory endpoints included the percentage changes from baseline in periprosthetic BMDs in Gruen zones 1 to 6 and over the entire periprosthetic region (zones 1 to 7) as well as the time‐related change in the translational and rotatory migration of the femoral stem at weeks 12, 22, and 48. Changes in BMDs of the contralateral hip, lumbar spine, and distal radius were evaluated by repeated DXAs. Changes in serum levels of bone turnover markers—procollagen type 1 N‐terminal propeptide (PINP) and C‐terminal telopeptide of type 1 collagen (CTX)—were measured using immunoassay techniques (IDS‐iSYS Intact PINP and IDS‐iSYS CTX‐I [CrossLaps], respectively) (Immonodiagnostic Systems Holdings PLC, Boldon, UK). The mean coefficient of variation values of PINP and CTX analyses were 3.7% and 1.7%, respectively. Evaluation of functional recovery included the measurements of walking speed^48^ using a validated gait analysis system (RehaWatch, Hasomed GmbH, Germany).^49^ Subjects were asked to walk at a self‐selected comfortable walking speed^48^ along a 10‐m walkway, and the mean coefficient of variation of the repeated measurements was 4.7%. Assessment of interindividual differences in daily walking activity was performed by means of digital pedometers^50^ for 7 days. Harris hip score (HHS),^34^ Western Ontario and McMaster Universities Osteoarthritis Index (WOMAC),^51^ Rand‐36,^51^ and Brief Pain Inventory (BPI)^52^ were applied as patient‐reported outcome measures (PROMs). Using BPI‐Short Form, subjects were asked to rate their pain severity in a four‐item questionnaire and the interference of pain with daily activities in a seven‐item questionnaire. The mean score (range 0 to 10) for pain severity and the mean score (range 0 to 10) for the interference of pain with daily activities were recorded. Exploratory endpoints included radiographic assessment of stem osseointegration and resorptive changes of the calcar region, based on the criteria of Engh and colleagues.^53^, ^54^

The occurrence of adverse events (AEs), serious adverse events (SAEs), adverse drug reactions (ADRs), and surgical complications were recorded. AEs were defined as any untoward medical occurrences. SAE was defined as any significant medical event that required in‐patient hospitalization. ADR was defined as any untoward and unintended responses to the investigational product. This meant that a causal relationship between the investigation product and the event could not be ruled out.

### Extension study

The extension study was carried out at a minimum of 2 years after the last dose of denosumab. The reevaluation was performed because discontinuation of denosumab results in a transient increase of bone turnover markers above baseline and BMD decline to pretreatment levels.^55^, ^56^ The extension study included vertebral‐fracture assessment by means of DXA.^57^ Bone turnover markers were not measured because serum levels of PINP and CTX return to baseline within 24 months after treatment discontinuation.^56^ Genant's classification of vertebral fractures^58^ was applied. It is based on the measurements of reductions in anterior, middle, and posterior heights of the vertebral body and the classification for deformities of shape (wedge, biconcave, crush). Mild deformity (grade 1) is defined as height loss ≥20% and <25%, moderate deformity (grade 2) as height loss ≥25% and <40%, and severe deformity (grade 3) as height loss >40%. The participants also filled the Owestry low‐back pain questionnaire. The scores for all questions answered were summed to obtain the index (range 0 to 100), with low scores (0 to 20) indicating no or minimal disability.

### Power analysis and statistical analysis

The expected change of periprosthetic BMD of zone 7 in the placebo group from baseline to 48 weeks was about –21%.^19^ We calculated that with a power of 90% (α = 0.05) and a standard deviation of 7%, a minimum of 28 subjects were needed in each group to detect the expected 50% reduction of periprosthetic BMD. This reduction was considered as a clinically meaningful change resembling the difference of periprosthetic BMD (zone 7) between women with normal or low BMD.^19^ Accordingly, we planned to recruit 68 subjects (34 in each group).

The primary and secondary endpoints (the outcome measured at week 48) were analyzed using linear mixed‐effects models for repeated measures. The method was also applied to evaluate intergroup differences at each time point (12, 22, and 48 weeks) as the exploratory endpoints of the different outcome parameters. No data were excluded. As supportive analyses, the primary and secondary endpoints were analyzed adjusting for covariates of interest, including age, body mass index (BMI), preoperative hip and lumbar spine BMDs, canal flare index, stem size, and stem‐to‐canal fill ratio. The level of statistical significance was set at 0.05 (two‐tailed). All analyses were performed using SAS System version 9.4 (SAS Institute, Cary, NC, USA).

The frequency of missing data (the number of examinations shown in brackets) was 0% for preoperative and postoperative hip radiographs (*n* = 195), 2.3% for RSA (*n = *260), 2.1% for PROMs (*n* = 390 for each PROM), 0.9% for periprosthetic BMD (*n* = 325), 2.6% for lumbar spine BMD (*n* = 195), 3.6% for distal radius BMD (*n = *195), 17.4% for contralateral hip BMD (including patients with contralateral hip arthroplasty) (*n = *195), 3.8% for walking parameters (*n = *390), 6.5% for pedometer measurements (*n = *260), and 1.9% for serum marker measurements (*n = *260).

## Results

### Baseline characteristics

Demographics and clinical characteristics at baseline were comparable between the denosumab and placebo groups (Table 1). The number of subjects with normal or low BMD was balanced within the two groups (Table 1). There were three smokers in the denosumab group and none in the placebo group.

### Periprosthetic BMD

**Figure 3:**
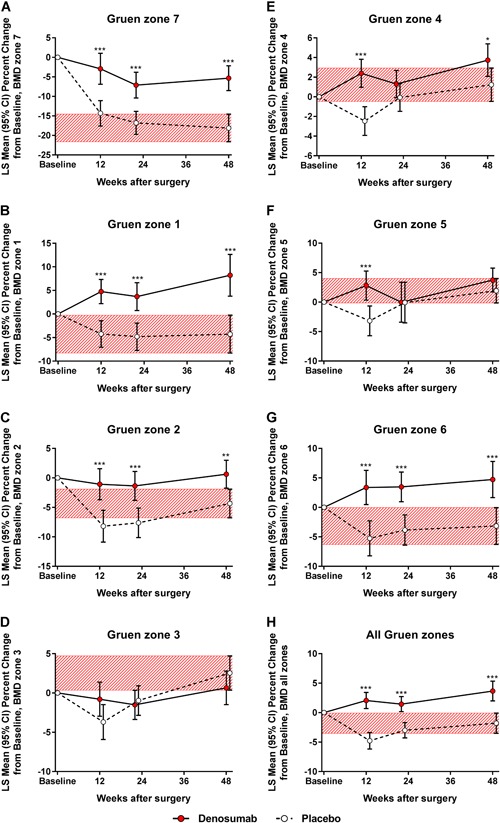
Least‐squares (LS) mean changes from baseline (and 95% confidence interval [CI]) for the periprosthetic bone mineral density (BMD) in the Gruen zones of the proximal femur. With respect to the primary endpoint, zone 7 (*A*), with respect to exploratory endpoints, zones 1 to 6 (*B–G*), and the entire prosthetic region (all zones combined) (*H*). The red‐hatched zones represent the 95% CI values of the placebo‐treated subjects at week 48. A linear mixed‐effects model for repeated measures supplemented with intergroup comparison at each postoperative time point was used for analysis (*p < 0.05, **p < 0.01, ***p < 0.001).

Denosumab significantly decreased bone resorption in the medial femoral neck (zone 7) (Fig. 3 *A*). Periprosthetic BMD of zone 7 decreased by 5.3% in the denosumab group and by 18.1% in the placebo group at week 48, with the primary efficacy (difference between the two groups) of 12.8% (95% confidence interval [CI] 8.2–17.4; *p* < 0.001).

With respect to exploratory endpoints (Fig. 3 *B–H*), denosumab increased periprosthetic BMD above the baseline in the greater (zone 1) and lesser trochanteric regions (zone 6) and in the entire periprosthetic region (*p* < 0.001 for all). Compared with the placebo group, the efficacy was 12.8% (95% CI 6.9–18.6) in the greater trochanteric region, 7.9% (95% CI 3.5–12.3) in the lesser trochanteric region, and 5.5% (95% CI 3.1–7.9) in the entire periprosthetic region at week 48. Adjustments for age, BMI, preoperative hip or lumbar spine BMD, canal flare index, stem size, and stem‐to‐canal fill ratio did not change the result. After discontinuation of denosumab, the periprosthetic BMDs of the denosumab group approached the levels of the placebo group by 3 years. Compared with baseline, periprosthetic BMD of the medial femoral neck (zone 7) was –12.6% (95% CI –18.5 to –6.6) in the denosumab group and –17.3% (95% CI –21.8 to –12.8) in the placebo group at 3 years.

### Femoral stem migration

**Figure 4:**
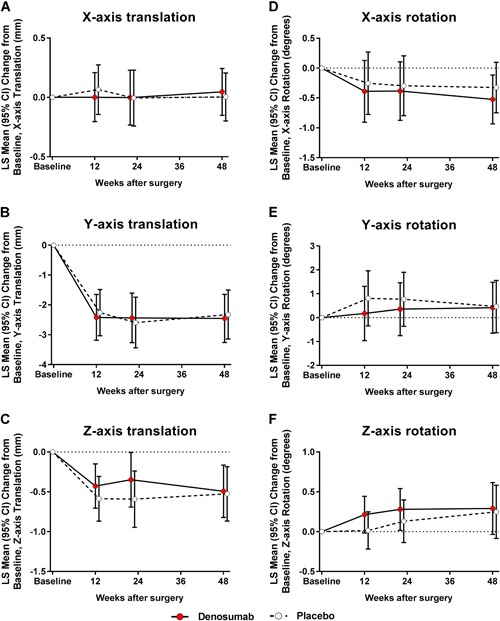
With respect to the secondary endpoint, the least‐squares (LS) mean changes from baseline (and 95% CI) were calculated for the femoral stem migration in translation along *x* axis (*A*), *y* axis (*B*), and *z* axis (*C*) and in rotation around *x* axis (*D*), *y* axis (*E*), and *z* axis (*F*). The intergroup differences at each postoperative time point were insignificant.

The two groups showed no significant differences in the amount of translation along the longitudinal *y* axis (stem subsidence) at week 48. Exploratory analyses of other translations as well as rotations around all axes showed no significant intergroup differences (Fig. 4 *A–F*). In both groups, translation and rotation occurred mainly during the first 12 weeks. The intergroup differences remained insignificant even after adjustments for age, BMI, local BMD, systemic *T*‐score, canal flare index, stem size, and stem‐to‐canal fill ratio.

### Functional recovery and patient‐reported outcome measures

**Figure 5:**
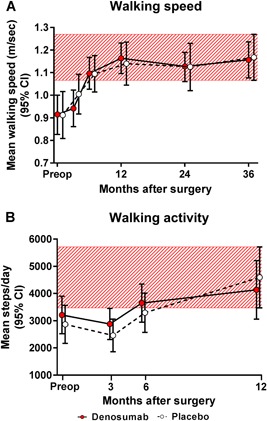
With respect to endpoints of functional recovery, the mean values (and 95% CI) of walking speed (*A*) and walking activity (*B*) were calculated. The red‐hatched zones represent the 95% CI values of the placebo‐treated subjects at the latest postoperative visit. The intergroup differences were insignificant.

**Figure 6:**
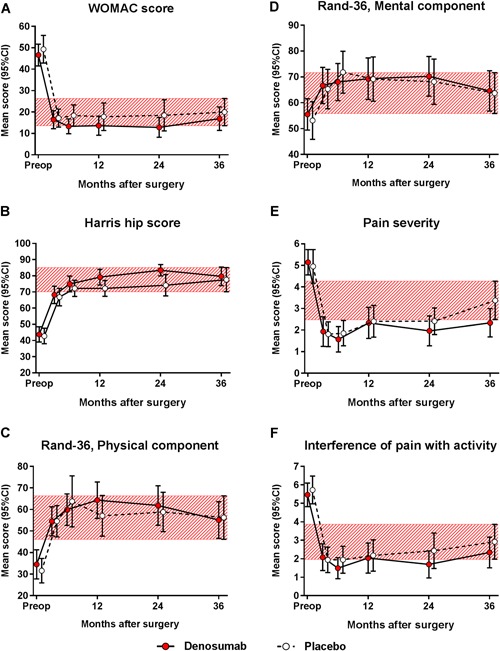
With respect to exploratory endpoint of clinical outcome using patient‐reported outcome measures, the mean scores (and 95% CI) of Western Ontario and McMaster Universities Osteoarthritis Index (WOMAC) (*A*), Harris hip score (*B*), Rand‐36 physical component (*C*), Rand‐36 mental component (*D*), Brief Pain Inventory (BPI) of pain severity (*E*), and BPI interference of pain with daily activities (*F*) were calculated. The red‐hatched zones represent the 95% CI values of the placebo‐treated subjects at the latest postoperative visit. The intergroup differences were insignificant.

The denosumab and placebo groups showed no statistical differences in walking speed and walking activity (Fig. 5 *A, B*). Preoperatively, 71% of the subjects had a walking speed below the critical level of 1.1 m/s.^59^ The walking speed improved by 0.25 m/s (95% CI 0.17–0.33) in the denosumab group and by 0.23 m/s (95% CI 0.12–0.34) in the placebo group by week 48. All PROMs improved compared with the preoperative values and did not differ between the two groups (Fig. 6 *A–F*).

### Serum markers of bone turnover and BMD changes

**Figure 7:**
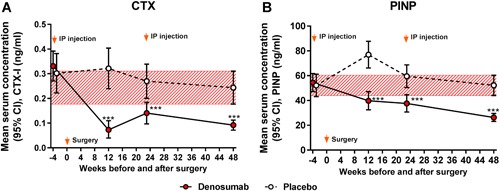
With respect to exploratory endpoint of antiresorptive efficacy of denosumab, the mean serum concentrations of bone‐resorption marker CTX (*A*) and bone‐formation marker PINP (*B*) were calculated. The red‐hatched zones represent the 95% CI values of the placebo‐treated subjects at week 48. The analysis employed a linear mixed‐effects model for repeated measures supplemented with intergroup comparison at each postoperative time point (***p < 0.001).

The serum level of the bone‐resorption marker CTX decreased rapidly by 77% in the denosumab group by week 12 (Fig. 7 *A*), with a concomitant 24% decrease of the bone‐formation PINP (Fig. 7 *B*). Bone turnover markers remained low in the denosumab group at all postoperative time points (*p* < 0.001 compared with the placebo group).

**Figure 8:**
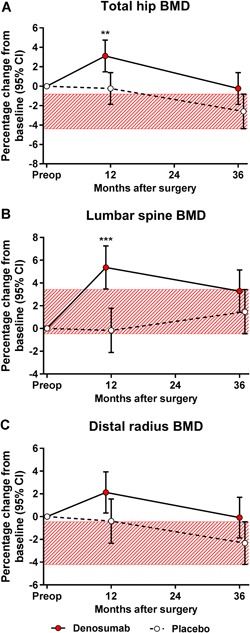
With respect to exploratory endpoint of systemic bone mineral density (BMD) response to denosumab, the mean percentage change from baseline (and 95% CI) for the total hip BMD (*A*), lumbar spine BMD (*B*), and distal radius BMD (*C*) were calculated. The red‐hatched zones represent the 95% CI values of the placebo‐treated subjects at 3 years. The analysis employed a linear mixed‐effects model for repeated measures supplemented with intergroup comparison at each postoperative time point (**p < 0.01, **p < 0.001).

Compared with the placebo group, denosumab increased the contralateral total hip BMD by 3.4% (95% CI 1.0–5.7; *p* = 0.005) (Fig. 8 *A*) and the lumbar spine BMD by 5.5% (95% CI 2.8–8.2; *p* < 0.001) (Fig. 8 *B*). BMDs decreased in response to discontinuation of denosumab, but the mean values remained at the upper CI levels of the placebo group (Fig. 8 *A–C*).

### Radiographic evaluation

At two years, all of the stems were classified as stable and osseointegrated according to the fixation and stability score^54^ (Table 2). The two groups did not differ in radiographic grading of bone resorption of the calcar.^53^ There were no cases with cortical thinning extending below the lesser trochanter into the diaphysis.

**Table 2:** Radiographic Evaluation of Stem Osseointegration at 2 Years

	Denosumab (*n = *33)	Placebo (*n* = 32)	*p* Value
Fixation score^a^ (mean ± SD)	8.2 ± 3.3	9.1 ± 2.5	0.226
Stability score^a^ (mean ± SD)	8.3 ± 1.5	8.7 ± 1.4	0.341
Endosteal bone bridging (no. [%])	24 (73)	27 (84)	0.367
Stable distal stem with pedestal formation (no. [%])	20 (61)	26 (81)	0.067
Thin (<1–2 mm) radiodense lines surrounding the distal stem (no. [%])	21 (64)	20 (63)	0.924
Distal cortical hypertrophy (no. [%])	12 (36)	10 (31)	0.663

For continuous data, *p* values are from two independent samples Mann‐Whitney *U* test. For categorical variables, *p* values are from chi‐square test or Fisher's exact test.

^a^ Mean score on a scale of 0 to 10.

### Safety endpoints and surgical complications

The incidence of AEs and SAEs was balanced in the two groups. During the 1‐year trial period, the number of AEs was similar in the denosumab and placebo groups (*n* = 40 and *n* = 43, respectively). The most common AE was low‐back pain. Five participants in the denosumab group and two participants in the placebo group were affected by SAE. No event showed a causal relationship to denosumab. No event was adjudicated as osteonecrosis of the jaw, atypical femur fracture, or clinical vertebral fracture. None of the subjects experienced periprosthetic infection, postoperative dislocation, or periprosthetic fracture.

As a registered adverse event, low‐back pain affected 19 subjects of the denosumab group (58%) and 12 subjects of the placebo group (38%) during the first 2 years after surgery. After discontinuation of denosumab, there were no intergroup differences in severity of low‐back pain and/or interference of low‐back pain with daily activities. Based on an increase of ≥2 BPI points (minimally clinically important difference), five subjects in the denosumab group and seven in the placebo group suffered from worsening of pain between 1 and 3 years after surgery. Based on the Owestry disability index score evaluated at 3 years, seven subjects from both groups suffered from moderate disability (score 21 to 40) and two subjects from both groups suffered from severe disability (score 41 to 60). Magnetic resonance imaging of the lumbar spine was performed in 24 subjects (13 in the denosumab group and 11 in the placebo group) because of radiating low‐back pain. Eight subjects in the denosumab group and three in the placebo group had severe lumbar disc degeneration. Five subjects in the denosumab group and eight in the placebo group had relative lumbar spine canal stenosis. One subject from both groups underwent decompressive lumbar spine canal surgery.

Based on vertebral fracture assessment by means of DXA performed during the off‐treatment period, four subjects in the denosumab group and five in the placebo group had one deformed vertebra. Three subjects in the denosumab group and two in the placebo group had multiple (two or three) deformed vertebrae. Five subjects in the denosumab group and four in the placebo group had a grade I deformity (mild crush or wedge). Grade 2 deformity (moderate crush or wedge) was found in six subjects of both groups.

## Discussion

As of now, denosumab is the most powerful inhibitor of osteoclastic activity, with near‐maximal reductions of bone resorption within days.^60^ Denosumab increased bone mineral density in the greater and lesser trochanters as well as in the entire periprosthetic region above baseline levels. This type of strong response has not been observed in trials with oral risedronate^18^ or intravenous zoledronic acid.^16^ The successful inhibition of periprosthetic bone resorption had no detectable effect on femoral stem migration; these findings are in line with the results of our study on zoledronic acid,^16^ which had no effect on migration of a double‐tapered straight femoral stem in postmenopausal women.

The impaired quality of cortical bone, including endosteal trabeculation and increased intracortical porosity of the proximal femur,^9^ seems to dictate the stability of uncemented femoral stems in aging women. Denosumab, if started 1 month before surgery, is probably too late in prevention of stem migration. We hypothesize that if denosumab treatment had been started early enough (such as 6 to 12 months before surgery), the cortical bone structure might have had time to respond and improve stem stability. Blocking of osteoclastic activity by means of denosumab decreases initial migration of cemented knee prosthesis,^61^ probably because of the predominance of trabecular bone at the implant‐bone interface in the knee region. The treatment response to denosumab is faster in trabecular bone than in cortical bone.^26^

All subjects of our trial received vitamin D and calcium supplementation because denosumab may cause hypocalcemia as an adverse event. For unknown reasons, total hip arthroplasty may also cause temporary asymptomatic postoperative hypocalcemia.^16^ On the other hand, vitamin D deficiency may impair osteoblastic functions.^62^, ^63^ Thus, it is possible that vitamin D deficiency could adversely affect biologic implant osseointegration and the quality of periprosthetic bone.

Parallel‐sided femoral stems, designed to engage metaphyseal cortical bone in the medial‐lateral plane,^37^ require adequate bone stock and unaltered femoral geometry.^11^ The stem allowed minor subsidence (1.5 mm, 95% CI 0.1−2.9) even in women with normal BMD. Although this migration was not clinically detrimental, it demonstrates the challenges to obtain secure press‐fit fixation. Self‐locking tapered femoral stems have been designed to accommodate a certain amount of migration to increase wedge fixation for the biologic process of bone ingrowth.^10^ The stem migration does not seem to start with the first steps of postoperative weight‐bearing but only after 1 week of mobilization.^64^ The degree of early weight‐bearing (unrestricted versus partial weight‐bearing) does not change the migration pattern.^64^ The current trial included gait analysis and the assessment of walking activity because the amount of physiological loading is one of the potential confounding patient‐related factors dictating the final amount of stem migration. Although the limited initial migration does not seem to prevent osseointegration,^15^, ^16^ continuous subsidence 6 to 12 months after the operation could indicate increased risk of future revision.^65^ However, there are not enough published results to draw definite conclusions.^45^ The difference between RSA‐measurable stem migration and clinically significant stem migration remains incompletely defined. Without doubt, excessive subsidence may cause permanent functional disability and carries even a risk for failure of osseointegration.^66^

The estimation of sample size was based on the power analysis to detect a clinically meaningful difference in the periprosthetic BMD. Presumably, there was a sufficient power also in evaluation of the intergroup differences in stem migration as the secondary outcome. Because of the high accuracy and precision of RSA, clinical trials can be performed with small patient populations.^46^ The group sizes of the current trial were above the recommended group size (15 to 25 subjects per group) in the RSA trials.^46^ To confirm a sufficient power for the definitive statement on the stem migration, a post hoc power analysis (α = 0.05, β = 0.80) was performed using the actual data of the current trial on standard deviations (2.48 mm; *y* axis translation). A difference of subsidence larger than 1.75 mm would have been recognized as being statistically significant. Apparently, this is also a clinically significant stem migration, albeit only detectable by RSA. As an illustration of the assumed clinical relevance, the current trial would have shown a significant intergroup difference of stem subsidence, if the denosumab‐treated group had shown a minimal subsidence similar to that reported in middle‐aged men and women (<65 years).^67^, ^68^

Bisphosphonate users may have a decreased risk of needing hip arthroplasty revisions.^69^, ^70^, ^71^ However, there is no direct evidence of the clinical benefit of pharmaceutical interventions, for example, in prevention of periprosthetic fractures, which are a cause for concern when using cementless total hip arthroplasty.^72^ Paradoxically, there are unmatched challenges to execute powered long‐lasting (≥10 years) trials in arthroplasty patients. The current designs of uncemented femoral stems show a high rate (>97%) of osseointegration.^2^, ^73^ Late periprosthetic fractures around osseointegrated femoral stems seem to appear on the second decade after surgery caused by minor trauma,^74^ resembling the trauma mechanisms of fragility hip fractures. The calculated yearly incidence of late periprosthetic femoral fractures is also low (≤0.4%),^72^ similar to fragility hip fractures (~0.4% to 1.0%).^75^ These facts explain why the current trial was not designed to study the impact of denosumab in prevention of periprosthetic fractures or mechanical loosening. Indeed, our trial faced no periprosthetic fractures or revision arthroplasties for other reasons during the 3‐year follow‐up.

In this randomized trial, denosumab increased periprosthetic femoral BMD, but this expected treatment response had no distinct effect on stem migration and speed of functional recovery. Future studies are needed for optimizing the preoperative timing of denosumab administration and for exploring efficient aftertreatment. The extension study confirmed that the action of denosumab is reversible.^26^ The discontinuation of denosumab caused no adverse events. The maintenance of treatment response and prevention of a rebound increase in bone resorption call for efficient aftertreatment protocols,^55^, ^76^ such as the single infusion of long‐lasting zoledronic acid.^16^ Before clinical use of denosumab in hip arthroplasty patients, the execution of large clinical trials should be focused on clinically relevant endpoints.

## Disclosures

HTA has received institutional research grants (Amgen) and is a member of an advisory scientific board (Amgen). All other authors state that they have no conflicts of interest.
